# Characterising individuals with newly diagnosed HIV before versus during the era of PrEP: a descriptive time-series analysis of data from a sexual health centre in Amsterdam, the Netherlands, 2015 to 2024

**DOI:** 10.2807/1560-7917.ES.2026.31.23.2500769

**Published:** 2026-06-11

**Authors:** Anders C Boyd, Eline S Wijstma, Feline de la Court, Mark AM van den Elshout, Kenneth Yap, Birgit van Benthem, Elske Hoornenborg, Maria Prins

**Affiliations:** 1 Department of Infectious Diseases, Public Health Service of Amsterdam, Amsterdam, the Netherlands; 2 Stichting hiv monitoring, Amsterdam, the Netherlands; 3 Amsterdam UMC location University of Amsterdam, Infectious Diseases, Amsterdam, the Netherlands; 4 Amsterdam Institute for Infection and Immunity, Infectious Diseases, Amsterdam, the Netherlands; 5 Centre for Infectious Disease Control, National Institute for Public Health and the Environment (RIVM), Bilthoven, the Netherlands; 6 Department of Infectious Diseases, Amsterdam UMC, University of Amsterdam, Amsterdam, the Netherlands; * These authors contributed equally to this work and share first authorship.

**Keywords:** HIV, pre-exposure prophylaxis for HIV, epidemiology, men who have sex with men, transgender, sex work

## Abstract

**BACKGROUND:**

With widespread HIV pre-exposure prophylaxis (PrEP) implementation, the population with increased chance of HIV acquisition may have shifted.

**AIM:**

This study aimed to explore the characteristics of people diagnosed with HIV in Amsterdam, the Netherlands before and during the national PrEP programme (NPP).

**METHODS:**

Using data from the Public Health Service of Amsterdam, we included 647 individuals diagnosed with HIV between 1 January 2015 and 30 June 2024. Sociodemographic, sexual and PrEP-related characteristics collected at diagnosis were analysed cross-sectionally. We used multivariable logistic regression to assess determinants of being diagnosed with HIV during (1 August 2019–30 June 2024) vs before the NPP (1 January 2015–31 July 2019).

**RESULTS:**

The number of newly diagnosed HIV infections decreased from 425 before the NPP(range: 92–103 diagnoses/year) to 222 during the NPP(range: 34–52 diagnoses per year). In multivariable analysis, transgender and gender-diverse persons (aOR = 12.74; 95% CI: 2.84–57.11) and individuals who engaged in sex work (aOR = 4.88; 95% CI: 2.67–8.91) or condomless sex (aOR = 1.95; 95% CI: 1.15–3.30) in the 6 months before diagnosis were more likely to be diagnosed during than before the NPP. Of 270 individuals with available data, 42 (15.6%) had used PrEP before their diagnosis.

**CONCLUSIONS:**

In the period after NPP implementation, the proportion of individuals diagnosed with HIV who were transgender or gender-diverse, engaged in sex work, or had condomless sex increased. Accessibility of PrEP services needs to be improved and timely PrEP uptake facilitated among specific subpopulations.

**Key public health message**

**What did you want to address in this study and why?**

Some populations may have benefitted more from the implementation of oral HIV pre-exposure prophylaxis (PrEP) than others. We aimed to understand which populations remain at risk of HIV infection despite PrEP implementation. This could help tailor and target PrEP programmes to more effectively support underserved populations.

**What have we learnt from this study?**

The number of new HIV diagnoses at the sexual health clinic in Amsterdam was lower in the PrEP era (August 2019–2024) than before (2015–July 2019). Decreases were especially stark among men who have sex with men and people with European ethnicity. The number of new HIV diagnoses among transgender or gender-diverse persons and sex workers was higher in the PrEP-era. Most individuals diagnosed with HIV were new to the clinic.

**What are the implications of your findings for public health?**

Thus far, subpopulations such as transgender persons and sex workers have probably benefitted less from oral PrEP implementation than other populations. Enhancing the accessibility of HIV prevention—for example through tailored PrEP provision in community-based settings, and targeted outreach—is crucial to ensure that all populations can benefit equitably from PrEP.

## Introduction

Pre-exposure prophylaxis (PrEP) is highly effective for preventing human immunodeficiency virus (HIV) acquisition and is available as an oral formulation in many high-income countries [1]. In countries where PrEP has been implemented, such as France [2], Germany [3], the Netherlands [4], and the United Kingdom (UK) [5], the incidence of HIV has declined. Nevertheless, new HIV infections continue to occur in these settings [1,6], suggesting that certain populations do not fully benefit from these PrEP programmes.

In the Netherlands, HIV has mainly affected men who have sex with men (MSM); cisgender women and cisgender heterosexual men made up roughly 37% of people living with HIV and 37% of people newly diagnosed with HIV in 2024 [7]. In the Netherlands, PrEP first became available in 2015 to a limited number of individuals enrolled in implementation studies [8]. Following European approval of oral tenofovir-emtricitabine for PrEP, some general practitioners and HIV treatment physicians started to prescribe PrEP, which the recipient had to pay out-of-pocket. From August 2019 to July 2024, PrEP access was expanded through a subsidised national PrEP pilot (NPP). The NPP was capped at 8,500 individuals nationally (including maximum 2,900 individuals in Amsterdam), while demand for PrEP was substantially higher, leading to long waiting lists [9]. Meanwhile, some general practitioners expressed reticence to providing PrEP, creating a barrier to accessing PrEP outside the NPP [10]. Access to PrEP was further constrained by personal, structural and socioeconomic factors [11,12].

The changes in the HIV prevention landscape following NPP implementation in 2019 may have led to a shift in the population that remains at risk of acquiring HIV. Characterising individuals diagnosed with HIV in the era of PrEP, compared with the period before, could help understand which populations benefited the most from PrEP and which groups remain at risk of HIV infection. In addition, identifying the characteristics of those who acquired HIV and had prior PrEP use could give insight into the subgroups who may face challenges in adhering to or maintaining PrEP use.

This study aimed to explore the sociodemographic characteristics, sexual behaviours and PrEP eligibility of those diagnosed with HIV in Amsterdam, the Netherlands between 2015 and 2024. More specifically, we compared the characteristics between those diagnosed with HIV during vs before the NPP and describe characteristics of individuals with vs without PrEP use in the year before their HIV diagnosis.

## Methods

### Study design, setting, and population

We conducted a descriptive, time-series analysis in which we summarised the characteristics of people newly diagnosed with HIV as cross-sectional time points. These time points were then merged for analysis to compare the characteristics of individuals with a new HIV diagnosis (i) during vs before the NPP and (ii) with vs without PrEP use. We used data from the centre for sexual health of the Public Health Service of Amsterdam (PHSA). In this study, we included all individuals newly diagnosed with HIV between 1 January 2015 and 30 June 2024 (near the end of the pilot programme).

The PHSA provides free-of-charge sexually transmitted infection (STI)/HIV testing and STI care to several key populations, including people with symptoms of STIs, individuals notified for an STI or HIV by a sexual partner, MSM, people younger than 25 years, transgender or gender-diverse (TGD) persons and sex workers [13]. Migrants have access to PHSA services, including PrEP, regardless of their legal status. The PHSA also offers PrEP tablets and PrEP care for eligible individuals. Since 2015, PrEP has been provided at a small scale through demonstration studies and clinical trials. From 2019 to 2024, the PHSA was allowed to provide PrEP care to a maximum of 2,900 individuals through the NPP; after this maximum capacity was reached in 2021, PrEP-eligible individuals could only enter the NPP if enrolled participants exited the programme or were lost to follow-up [8,14-16]. In the NPP, individuals were required to pay a small co-pay of EUR 7.50 for 30 tablets, and all other care was free-of-charge. The 2024 Dutch PrEP eligibility criteria pertain to MSM or TGD persons who have, in the past 6 months, had (i) condomless anal sex with a male partner with unknown HIV status or a known HIV-positive partner with a detectable viral load, (ii) an anal STI or syphilis, (iii) used post-exposure prophylaxis (PEP), or (iv) engaged in chemsex; others (including cisgender women and heterosexual men) can be eligible based on individual circumstances [16]. Eligibility is assessed during the intake visit. Throughout the NPP, the PHSA prioritised certain subpopulations for enrolment (i.e. TGD persons, sex workers, uninsured persons, individuals who recently migrated to the Netherlands, and those younger than 25 years) [9].

### Data collection and study variables

At the PHSA, healthcare workers collect data in electronic patient files at each visit. Collected data include sociodemographic characteristics, sexual behaviour, STI and HIV testing and diagnosis, and PrEP use. All data are coded, secured and pseudonymised in accordance with Dutch privacy legislation. Visitors consent that pseudonymised data can be used for monitoring, surveillance and research through an opt-out procedure.

Using data from the visit where HIV was diagnosed, we extracted information on the following sociodemographic variables: age, gender and sexuality group (i.e. MSM, TGD persons, cisgender heterosexual men and cisgender women), education level (i.e. primary, secondary, tertiary or unspecified), country of birth (the Netherlands or other), and ethnicity (Dutch, non-Dutch European, Central or South American, African, Asian, other; taking into account the countries of birth of the client and their parents based on the categorisation algorithm of Statistics Netherlands [17]). Sexual behaviour variables included: any condomless sex (yes/no; and if yes, specified for insertive anal, receptive anal, or vaginal sex, stratified by partner type: casual/steady), number of sex partners, any sex in exchange for money or goods (herein referred to as sex work), and drug or alcohol use during sex; all pertaining to the 6 months before diagnosis. We categorised sexualised drug use as: none, sexualised drug use with chemsex (defined as any self-reported sexualised use of methamphetamine, γ-hydroxybutyric acid/γ-butyrolactone, mephedrone, ketamine, amphetamine or ecstasy) or sexualised drug use without chemsex (defined as any self-reported sexualised use of drugs excluding the abovementioned chemical drugs). An STI diagnosis refers to a chlamydia, gonorrhoea and/or syphilis infection diagnosed at the visit of HIV diagnosis. We also extracted self-reported information on PrEP use via the NPP or elsewhere. These data were only collected from 7 May 2018 onward from MSM and TGD persons with a previous HIV-negative test at the PHSA or elsewhere. The PrEP-related data included any PrEP use (i.e. at least one dose of PrEP) in the year preceding HIV diagnosis and, for those with PrEP use, PrEP regimen (i.e. daily or event-driven) and source (i.e. PHSA, general practitioner, HIV specialist, online, PrEP demonstration project, abroad/other).

Based on 2024 PrEP eligibility criteria and available data, we defined PrEP eligibility as having at least one of the following in the previous 6 months: any condomless anal sex with a casual partner, an anal STI diagnosis or syphilis, or chemsex. Data on HIV status of sex partners or HIV RNA viral load of HIV-positive partners were not available; we assumed that the HIV status of a casual partner was unknown. Data on prior PEP use were also not available.

We extracted data on prior PHSA visits (from 2013 onward) and, if applicable, the date of the last negative HIV test. For individuals without a prior PHSA visit, we used the self-reported date of their last negative test. We calculated the time between the last negative HIV test and diagnosis as < 1 year, 1–2 years, or > 2 years. If available, we extracted Fiebig stage at diagnosis. A recent HIV diagnosis was defined as a diagnosis < 1 year after the last negative test or at Fiebig stage I–V.

### Statistical analysis

We descriptively summarised sociodemographic characteristics, sexual behaviour and PrEP-related variables of individuals diagnosed with HIV. We report the number of individuals tested for HIV and HIV tests performed by year and period.

We first compared the characteristics of those diagnosed with HIV during the NPP (1 August 2019–30 June 2024) with those diagnosed before the NPP (1 January 2015–31 July 2019). Second, among those diagnosed with HIV between 7 May 2018 and 30 June 2024, we compared the characteristics of those who used PrEP against those who did not use PrEP in the year before their HIV diagnosis. We assumed that cisgender heterosexual men and cisgender women without information on prior use had not used PrEP, given the extremely low PrEP uptake in these populations in the Netherlands [13]. We compared these characteristics using Pearson’s chi-square or Fisher’s exact test for categorical variables and the rank sum test or Mann–Whitney U test for continuous variables.

We then modelled the probability of having a HIV diagnosis during (vs before) the NPP using logistic regression. We calculated univariable odds ratios (OR) and 95% confidence intervals (CI) comparing the odds across levels of determinants by adding covariates of various sociodemographic characteristics and sexual behaviours to the model. We then constructed a multivariable logistic regression model by adding the following covariables a priori, due to their hypothesised relevance to our outcomes: age, gender and sexuality group, ethnicity, education (at the tertiary level), condomless sex, sex work and STI diagnosed at visit of HIV diagnosis.

For 10 cisgender men, information on sexual preference and partner type was missing, and we assumed they were MSM. For all other covariables with missing values, we used multiple imputation by chained equations to impute missing values based on logistic regression. In the imputation model, imputed variables (all binary or recoded as binary) were tertiary education level, any condomless sex, chemsex and sex work. Predictor variables were age, gender and sexuality group, ethnicity, and any STI diagnosed at visit of HIV diagnosis. We created 20 imputed datasets, and parameter estimates were pooled using Rubin’s rules. We did not model the covariable chemsex because data were mostly unavailable before the NPP.

In a sensitivity analysis, we included condomless anal sex with a casual partner, instead of condomless sex, in the multivariable model to assess the specific contribution of this behaviour to HIV diagnoses during the NPP.

Statistical significance was considered at p < 0.05. Analyses were performed using STATA (v17, College Station, United States).

## Results

### Study population characteristics

Between 1 January 2015 and 30 June 2024, 647 individuals were diagnosed with HIV at the PHSA (Table 1). Most (n = 536; 82.8%) were MSM and 42 (6.5%) were TGD. Median age was 31 years (interquartile range (IQR): 26–39). Nearly half (n = 283; 43.7%) had European ethnicity and 35.9% (n = 232) were born in the Netherlands. The majority (472; 73.0%) reported condomless sex in the 6 months preceding their HIV diagnosis, 41.6% (n = 269) reported condomless anal sex with a casual partner, and 15.3% (n = 99) reported sex work (Table 1). At the HIV diagnosis visit, 41.3% (n = 267) had a concomitant bacterial STI diagnosis, and 62.8% (n = 406) met at least one PrEP-eligibility criterion (Table 1).

**Table 1:** Characteristics of people diagnosed with HIV at the Public Health Service of Amsterdam, stratified by diagnosis before or during the national PrEP programme, the Netherlands, 2015–2024 (n = 647)

	Total n = 647		Before the NPP ^a^ n = 425		During the NPP ^a^ n = 222		p value^b^
	Median or n	IQR or %	Median or n	IQR or %	Median or n	IQR or %	
Sociodemographic characteristics							
**Age in years**	31	26–39	32	26–41	31	26–37	0.16
**Sexual group**							
MSM	536	82.8	370	87.1	166	74.8	< 0.001
TGD person^c^	42	6.5	5	1.2	37	16.7	
Cisgender heterosexual man	26	4.0	16	3.8	10	4.5	
Cisgender woman	33	5.1	25	5.9	8	3.6	
Missing	10	1.6	9	2.1	1	0.5	
**Education level**							
Tertiary education^d^	272	42.0	186	43.8	86	38.7	0.0052
Senior secondary education^e^	113	17.5	86	20.2	27	12.2	
Foundational education^f^	63	9.7	37	8.7	26	11.7	
Other^g^	158	24.4	88	20.7	70	31.5	
Missing	41	6.4	28	6.6	13	5.9	
**Born in the Netherlands **(4 missing)	232	35.9	176	41.4	56	25.2	< 0.001
**Ethnicity^h^**							
European	283	43.7	205	48.2	78	35.1	< 0.001
Central/South America	221	34.2	121	28.5	100	45.1	
Africa	63	9.7	48	11.3	15	6.7	
Asia	67	10.4	43	10.1	24	10.8	
Other	13	2.0	8	1.9	5	2.3	
Sexual behaviour in the 6 months before diagnosis							
**Condomless sex**							
Any (64 missing)	472	73.0	294	69.2	178	80.2	0.21
Any with a casual partner (5 missing)	287	44.4	155	36.5	132	59.5	< 0.001
**Condomless anal sex**							
Any (65 missing)	439	67.9	273	64.2	166	74.8	0.022
Any with a casual partner (7 missing)	269	41.6	147	34.6	122	55.0	< 0.001
**Condomless vaginal sex**							
Any (56 missing)	46	7.8	28	7.0	18	9.5	0.29
Any with a casual partner (30 missing)	20	3.2	8	1.9	12	6.2	0.012
**Number of sex partners**	6	3–15	5	2–12	7	3–30	0.0058
Missing	21	3.2	16	3.8	5	2.3	
**Sex work**	99	15.3	28	6.6	71	32.0	< 0.001
Missing	26	4.0	19	4.5	7	3.2	
STIs and PrEP-eligibility at HIV diagnosis visit							
Any STI	267	41.3	184	43.2	83	37.4	0.15
Chlamydia	146	22.6	103	24.2	43	19.4	0.16
Gonorrhoea	143	22.1	100	23.5	43	19.4	0.23
Infectious syphilis (stage I, II, recent latent)	57	8.8	33	7.8	24	10.8	0.19
PrEP-eligible^i^	406	62.8	248	58.4	158	71.2	0.0014
HIV testing and diagnosis							
Recent HIV infection, based on Fiebig stage and date of last negative HIV test	275	43.0	186	43.8	89	40.1	0.27
Unknown	8	1.2	8	1.9	0	0.0	
**Fiebig stage at diagnosis^j^**							
I–V	55	26.3	12	29.3	43	25.6	0.63
VI	154	73.7	29	70.7	125	74.4	
Any prior visit to the PHSA (2013 onwards)	280	43.3	196	46.1	84	37.8	0.044
**Time since last negative HIV test at PSHA (among n = 267 persons with a prior HIV test at the PHSA)^k^**							
< 1 year before diagnosis	185	69.7	138	73.8	47	58.8	< 0.001
1–2 years before diagnosis	42	15.7	32	17.1	10	12.5	
> 2 year before diagnosis	40	15.0	17	9.1	23	28.8	
**Self-reported HIV testing history (among n = 380 individuals without a prior HIV test at the PHSA)^k^**							
< 1 year before diagnosis	62	16.3	42	17.7	20	14.1	0.086
1–2 years before diagnosis	111	29.2	63	26.5	48	33.8	
> 2 year before diagnosis	115	30.3	82	34.5	33	23.2	
No prior HIV test	74	19.5	42	17.7	32	22.5	
Unknown	18	4.7	9	3.8	9	6.3	

HIV: human immunodeficiency virus; IQR: interquartile range; MSM: men who have sex with men; NPP: national PrEP programme; PHSA: Public Health Service of Amsterdam; PrEP: pre-exposure prophylaxis for HIV; STI: sexually transmitted infection; TGD: transgender or gender-diverse.

^a ^Before the NPP includes HIV diagnoses occurring between 1 January 2015 and 31 July 2019. During the NPP includes HIV diagnoses occurring between 1 August 2019 and 30 June 2024.

^b ^p values were calculated were based on non-missing data; For categorical variables, we used a Pearson’s chi-square test or Fisher’s exact test to test for differences. For continuous variables, we used a rank sum test or Mann–Whitney U test.

^c ^The TGD category included 35 transgender women and seven non-binary persons.

^d ^University or university of applied sciences.

^e ^Senior secondary vocational education, senior general secondary education, pre-university education.

^f ^Primary education, pre-vocational secondary education, secondary vocational education, special education or no education.

^g ^Including non-university education outside the Netherlands.

^h ^Following the classification of Statistics Netherlands [17], ethnicity was determined by the country of birth of the client (if the client was born abroad), the client’s father (if the client and mother were born in the Netherlands but the father was born abroad), or the client’s mother (if the client was born in the Netherlands but the mother or both parents were born abroad).The ‘other’ category includes people with North American and Oceanian ethnicity.

^i ^Criteria include any condomless sex with a casual partner, a rectal STI diagnosis or syphilis, or chemsex in the 6 months before the visit. Individuals with missing data on a given criterion were assumed to not have that criterion.

^j ^Fiebig stage was unknown for 438 individuals (n = 384 diagnosed before the NPP and n = 54 diagnosed during the NPP). No individuals were diagnosed with Fiebig stage I.

^k ^Thirteen individuals with a prior PHSA visit were not previously tested for HIV at the PHSA.

Additional data on intersectionality of gender and sexuality, migration background and sex work in individuals included in the analysis are appended in Supplementary Figure S1. The proportion born outside the Netherlands was highest among TGD persons (40/42), followed by cisgender women (26/32), cisgender heterosexual men (19/26) and MSM (325/534). The proportion reporting sex work was highest among TGD persons (34/41), followed by cisgender women (10/32) and MSM (55/522), and lowest among cisgender heterosexual men (0/26).

Most individuals (n = 367; 56.7%) were diagnosed with HIV at their first ever visit to the PHSA. An HIV diagnosis at the first PHSA visit occurred most frequently among cisgender women (28/33), TGD persons (35/42), and cisgender heterosexual men (19/26), less frequently among MSM (284/536; 53.0%). An HIV diagnosis at the first PHSA visit was more common during the NPP (138/222; 62.2%) than before (229/425; 53.9%; p = 0.044).

Of the 41.3% (n = 267) individuals who previously tested HIV-negative at the PHSA, 185 (69.3%) had been tested within a year of their diagnosis (Table 1). The proportion of individuals with a recent negative HIV test at the PHSA was lower during than before the NPP. Among 380 individuals without a previous HIV test at the PHSA, 62 (16.3%) self-reported testing HIV-negative within a year before their diagnosis, 111 (29.2%) within 1–2 years, and 115 (30.3%) > 2 years before; 92 (24.2%) reported having never had an HIV test. Among those with available data, the proportion with Fiebig stages I–V was comparable during and before the NPP (12/41; 29.3% and 43/168; 25.6%, respectively (Table 1). Additional data on HIV testing history by subpopulation are made available in Supplementary Table S1.

### HIV testing over time

**Figure 1:**
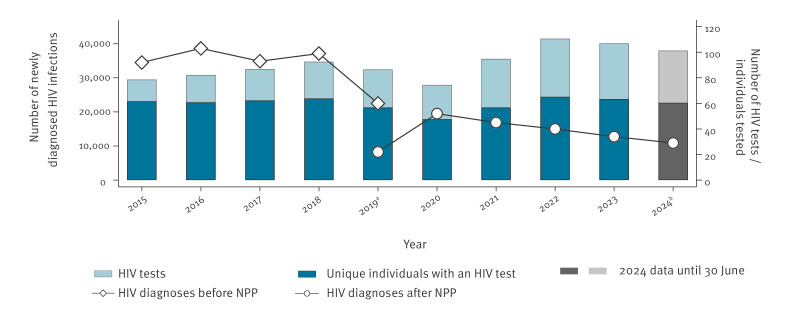
Newly diagnosed HIV infections, HIV tests, and individuals tested for HIV per year at the Public Health Service of Amsterdam, the Netherlands, 1 January 2015–30 June 2024

HIV: human immunodeficiency virus; NPP: national pre-exposure prophylaxis programme.

^a ^In 2019, before NPP refers the period between 1 January 2019 and 31 July 2019, and during NPP refers to the period between 1 August 2019 and 31 December 2019.

^b^ We included data until 30 June 2024.

**Figure 2:**
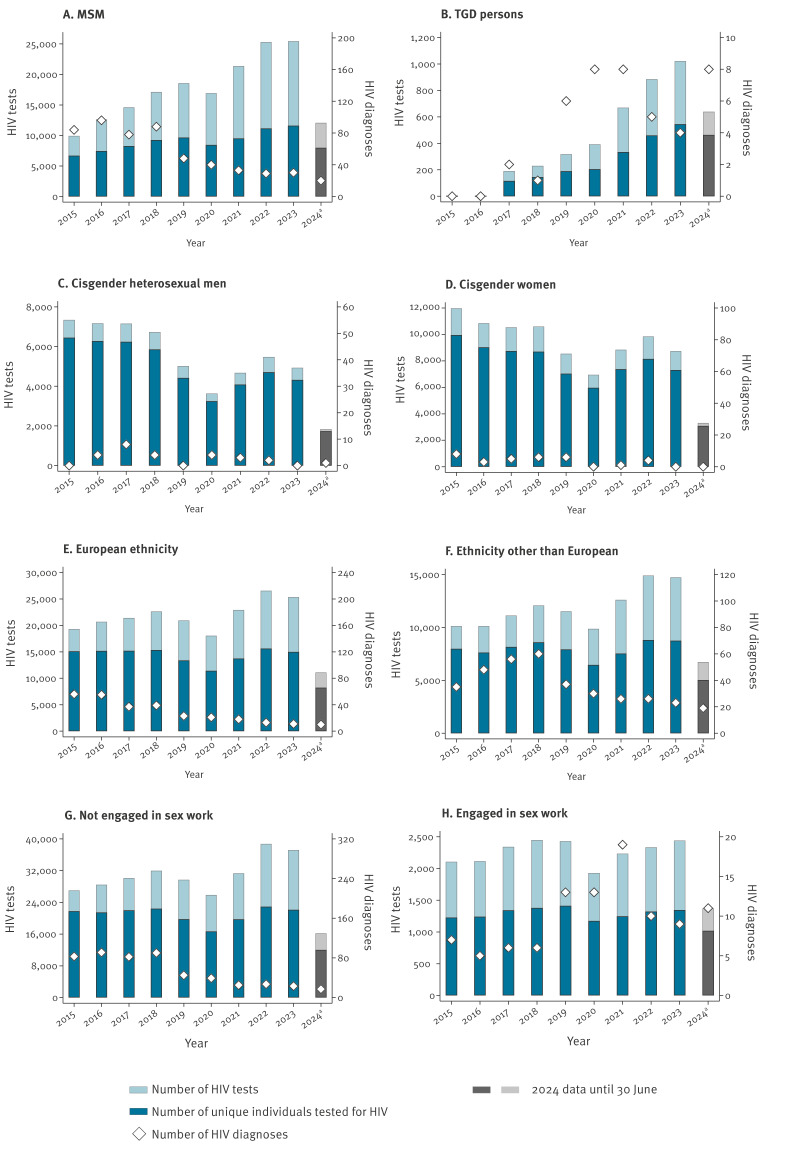
Newly diagnosed HIV infections, HIV tests, and individuals tested for HIV sociodemographic subpopulations per year at the Public Health Service of Amsterdam, the Netherlands, 1 January 2015–30 June 2024

HIV: human immunodeficiency virus; MSM: men who have sex with men; TGD: transgender or gender-diverse.

^a^ We included data until 30 June 2024.

The number of individuals tested for HIV at the PHSA remained stable around 23,000 tests per year, with a slight decrease in 2020 (17,853 individuals tested) during the COVID-19 pandemic (Figure 1). The number of HIV tests performed yearly increased from 29,507 in 2015 to 40,140 in 2023, in part due to quarterly HIV screening among PrEP users. Notably, we observed several shifts in the subpopulations tested for HIV. The number of individuals tested for HIV increased among TGD persons (from 170 in total before to 1,109 in total during the NPP) and among MSM (from 18,692 to 23,465), while it decreased among cisgender heterosexual men and cisgender women (Figure 2). The total number of sex workers tested for HIV remained relatively stable over time (Figure 2), but the number of sex workers with Central or South American ethnicity tested for HIV increased from 489 before to 1,073 during the NPP (with the yearly totals of sex workers included in this analysis stratified by ethnicity available in Supplementary Figure S3). Additional data on the number of HIV tests for different subpopulations per year are appended in Supplementary Table S2 and per period (i.e. during and before the NPP) in Supplementary Table S3.

There were 343,276 HIV tests among 122,599 unique individuals during the study period. Per year, the numbers of HIV tests and diagnoses at the PHSA were, respectively, 29,507 and 92 in 2015, 30,844 and 103 in 2016, 32,560 and 93 in 2017, 34,746 and 99 in 2018, 32,470 and 60 in 2019, 27,915 and 52 in 2020, 35,568 and 45 in 2021, 41,515 and 40 in 2022, and 40,140 and 34 in 2023.

In total, there were 343,276 HIV tests among 122,599 unique individuals during the study period. In each panel, both Y-scales are relative to the maximum numbers of HIV tests within a year.

### HIV diagnoses during and before the national pre-exposure prophylaxis programme

Most HIV diagnoses (425/647; 65.7%) occurred before the NPP and 222 (34.3%) occurred during the NPP. Before the NPP, the total number of new HIV diagnoses was stable over calendar years (range: 92–103). In 2019, the total number of new HIV diagnoses roughly halved compared with the previous years, and thereafter decreased every year, to 34 in 2023 (Figure 1).

Despite a decrease in the total number of HIV diagnoses, the absolute number of HIV diagnoses for TGD persons increased from five to 37 and for sex workers from 28 to 71 between the period before and during the NPP (Table 1). The proportions of cisgender heterosexual men and cisgender women were similar across periods, and the proportion of MSM was lower during vs before the NPP. The proportion of HIV diagnoses among people with Central or South American ethnicity was higher during the NPP. In multivariable analysis, TGD persons, individuals who engaged in sex work and individuals who engaged in condomless sex had higher odds of being diagnosed with HIV during vs before the NPP (Table 2). These results did not substantially change when including condomless anal sex with a casual partner in the multivariable model; the parameter estimates from this model are appended in Supplementary Table S4.

**Table 2:** Determinants of having an HIV diagnosis at the Public Health Service of Amsterdam during vs before the national PrEP programme, the Netherlands, 1 January 2015–30 June 2024 (n = 647)

	Univariable			Multivariable^a^		
	OR	95% CI	p	aOR	95% CI	p
Age (per year)	0.99	0.97–1.00	0.073	0.99	0.99–1.01	0.72
Sexual group^b^	NA		0.12^c^	NA		0.42^c^
MSM	Reference					
TGD person	16.79	6.49–43.49	NA	12.74	2.84–57.11	0.0010
Cisgender heterosexual man	1.42	0.63–3.19	NA	2.37	0.92–6.09	0.074
Cisgender woman	0.73	0.32–1.64	NA	0.70	0.26–1.88	0.49
Ethnicity^d^	NA		0.21^c^	NA		0.55^c^
European	Reference					
Central/South American	2.17	1.50–3.15	< 0.001	1.18	0.75–1.86	0.47
African	0.82	0.43–1.56	0.54	0.68	0.31–1.49	0.34
Asian	1.47	0.84–2.58	0.18	1.31	0.71–2.40	0.39
Other/unknown	1.64	0.52–5.17	0.40	1.54	0.41–5.80	0.52
Education level						
Tertiary education	0.80	0.57–1.12	0.20	1.45	0.96–2.19	0.079
Sexual behaviour and STIs						
Any condomless sex^e^ (yes vs no)	1.83	1.14 -2.94	0.12	1.95	1.15–3.30	0.013
Sex work^e^ (yes vs no)	6.58	4.08–10.61	< 0.001	4.88	2.67–8.91	< 0.001
Any STI diagnosed at HIV diagnosis visit (yes vs no)	0.78	0.56–1.09	0.15	0.74	0.50–1.09	0.13

aOR: adjusted odds ratio; CI: confidence interval; HIV: human immunodeficiency virus; OR: odds ratio; MSM: men who have sex with men; PHSA: Public Health Service of Amsterdam; STI: sexually transmitted infection; TGD: transgender or gender-diverse.

^a ^All odds ratios are adjusted by the variables presented in the table.

^b ^Ten cisgender men without available information on sexual preference were assumed to be MSM (4/10 were previously diagnosed with a rectal STI at the PHSA).

^c^ Overall p value.

^d ^Following the classification of Statistics Netherlands [17], ethnicity was determined by the country of birth of the client (if the client was born abroad), the client’s father (if the client and mother were born in the Netherlands but the father was born abroad), or the client’s mother (if the client was born in the Netherlands but the mother or both parents were born abroad). The ‘other’ category includes people with North American and Oceanian ethnicity.

^e^ Refers to 6 months before the visit.

All parameter estimates were calculated using logistic regression with multiple imputation.

### HIV diagnosis with vs without prior PrEP use

Data on PrEP use in the previous year was available for 270 individuals diagnosed in March 2018 or later; 42 (15.6%) reported using PrEP in the year before their HIV diagnosis, including 27 (10.0%) who reported using PrEP in the previous 3 months (Table 3). All users were MSM (n = 37) or TGD persons (n = 5). The majority (n = 25) used PrEP following an event-driven regimen. Most (n = 27) obtained PrEP formally in the Netherlands, while 12 individuals obtained it informally or abroad. Four of these 12 were diagnosed during their formal PrEP intake at the PHSA. Among those who did (vs did not) previously use PrEP, the median number of sex partners, the proportion reporting chemsex, and the proportion reporting condomless anal sex with a casual partner were higher (Table 3). Among people without a history of PrEP use, 20 individuals were diagnosed with HIV during their NPP intake visit; 14 of them were TGD or cisgender male sex workers at their first ever PHSA visit at the Amsterdam Center for Sex Workers.

**Table 3:** Characteristics of people diagnosed with HIV at the Public Health Service of Amsterdam, stratified by PrEP use, the Netherlands, 7 May 2018–30 June 2024 (n = 270)

	Total n = 270		No prior PrEP n = 228		Prior PrEP n = 42		p value
	Median or n	IQR or %	Median or n	IQR or %	Median or n	IQR or %	
Age in years	31	26–38	31	26–38	30	26–38	0.72
Sexual group							
MSM	203	75.2	166	72.8	37	88.1	0.085
TGD person	38	14.1	33	14.5	5	11.9	
Cisgender heterosexual man	12	4.4	12	5.3	0	0.0	
Cisgender heterosexual woman	17	6.3	17	7.5	0	0.0	
Education level (13 missing)							
Tertiary	111	43.2	89	41.4	22	52.4	0.19
Country of birth (2 missing)							
The Netherlands	75	28.0	62	27.4	13	31.0	0.64
Ethnicity							
European	99	36.7	84	36.8	15	35.7	0.61
Central/South American	116	43.0	96	42.1	20	47.6	
African	23	8.5	22	9.6	1	2.4	
Asian	27	10.0	22	9.6	5	11.9	
Other/unknown	5	1.9	4	1.8	1	2.4	
Sexual behaviour							
Any condomless sex^a ^(19 missing)	219	87.3	183	86.3	36	92.3	0.30
Condomless insertive anal sex (22 missing)	147	59.3	118	57.0	29	70.7	0.10
Condomless receptive anal sex (17 missing)	174	68.8	141	66.5	33	80.5	0.077
Condomless anal sex with a casual partner (2 missing)	148	55.2	118	52.2	30	71.4	0.021
Condomless vaginal sex (205 missing)	44	67.7	43	68.3	1	50.0	0.59
Number of sex partners^a^	7	3–20	6	2–20	10	5–40	0.036
Chemsex^a ^(18 missing)	59	23.4	45	20.8	14	38.9	0.018
Sex work^a^ (6 missing)	79	29.9	64	28.6	15	37.5	0.26
STI diagnosis at HIV diagnosis visit							
Any STI	103	38.1	84	36.8	19	45.2	0.30
Chlamydia	59	21.9	49	21.5	10	023.8	0.74
Gonorrhoea	53	19.6	42	18.4	11	26.2	0.24
Syphilis	27	10.0	21	9.2	6	14.3	0.31
PrEP eligible^b^							
Yes	191	70.7	154	67.5	37	88.1	0.007
Period of HIV diagnosis							
During NPP	217	80.4	183	80.3	34	81.0	0.92
Calendar year of HIV diagnosis^c^							
2018	17	6.3	12	5.3	5	11.9	0.008
2019	58	21.5	54	23.7	4	9.5	
2020	52	19.3	47	20.6	5	11.9	
2021	44	16.3	38	16.7	6	14.3	
2022	38	14.1	34	14.9	4	9.5	
2023	32	11.9	22	9.6%	10	23.8	
2024	29	10.7	21	9.2%	8	19.0	

HIV: human immunodeficiency virus; IQR: interquartile range; MSM: men who have sex with men; NPP: national PrEP programme; PrEP: pre-exposure prophylaxis for HIV; STI: sexually transmitted infection; TGD: transgender or gender-diverse.

^a^ Refers to the 6 months before the visit.

^b^ Criteria include any condomless sex with a casual partner, a rectal STI diagnosis or syphilis, or chemsex in the 6 months before the visit. Individuals with missing data on a given criterion were assumed not to have that criterion.

^c ^Incomplete calendar years with 2018 starting at 7 May 2018 and 2024 ending at 30 June 2024.

PrEP use refers to any reported use in the year before the visit at which participants were diagnosed with HIV. Data on PrEP use were first collected in 2018.

## Discussion

Among individuals attending the PHSA, new HIV diagnoses decreased between 2015 and 2024 despite expanded HIV testing. Simultaneously, there was an increase in the absolute numbers and proportions of HIV diagnoses among TGD persons and sex workers after implementation of the national PrEP programme, yet these totals remain small. The proportion of HIV diagnoses among individuals who engaged in condomless sex in the 6 months preceding their diagnosis also increased. Among individuals with available data on PrEP use, 16% had used PrEP in the year before their HIV diagnosis. These data provide insight into the changing HIV epidemic in Amsterdam with the wider accessibility of PrEP.

The increase in HIV diagnoses among TGD persons and sex workers is particularly noteworthy and warrants some explanation. It may suggest a rise in HIV incidence but could also reflect enhanced acknowledgement or engagement of these groups in HIV testing, particularly as recruitment efforts were focused on these populations during the NPP. The number of TGD persons tested for HIV was roughly six times higher during than before the NPP. This increase may reflect that more TGD persons disclosed their TGD identity over time as a result of greater societal visibility and implementation of the two-question method to ask about gender at the PHSA in 2017 (i.e. “What is your current gender identity?” and “What was your assigned sex at birth?”) [18]. Moreover, the PHSA has improved its capacity to engage TGD persons in sexual healthcare after opening the Trans United Clinic in 2021, offering integrated sexual health and gender-affirming care to marginalised TGD persons in a community-based setting [19]. Improved engagement in care may also explain some of the increase in HIV diagnoses among sex workers. Starting in 2015, the Amsterdam Center for Sex Workers (ACS) appointed Spanish- and Portuguese-speaking community representatives to its outreach team. This resulted in greater engagement of Central and South American sex workers both directly and indirectly through word-of-mouth within the community. Moreover, the ACS automatised their online outreach in 2021, which could have further improved engagement of TGD and cisgender male sex workers.

Nonetheless, the increases in HIV diagnoses among TGD persons and sex workers indicate missed opportunities for prevention. Roughly one in three individuals belonging to these groups had a (self-reported) negative HIV test within a year before their diagnosis, and roughly two in three within 2 years – indicating a window of opportunity for linkage to PrEP care. As such, we need a more thorough understanding of the barriers to HIV prevention (including PrEP) that these subpopulations are experiencing. Prior research has indicated that barriers exist on the structural (e.g. costs, waitlists, physical distance), interpersonal (e.g. stigma, culturally mismatched care) and individual (e.g. competing priorities, socioeconomic precariousness, insufficient knowledge) level [20-23]. Notably, tailored and integrated care, including PrEP care, is already being offered to sex workers at the ACS and the Amsterdam Trans Clinic; sex workers and TGD persons were also prioritised for NPP enrolment [9]. Yet, our findings indicate that these efforts may not be sufficient and further interventions are needed. For example, by increasing PrEP awareness in the community and among social workers serving these populations, more broadly integrating gender-affirming care into PrEP services, reducing stigma around sex work and PrEP, and adopting a policy whereby PrEP is offered to all who request it – eliminating the need the disclose one’s TGD identity or sex work status to access PrEP [20-23]. HIV diagnoses among cisgender women and heterosexual men were low despite relatively high testing volumes and did not notably change over time. Combined with national data showing frequent late-stage diagnosis and low PrEP uptake in these groups [7,13], these findings highlight the need for improved case-finding and more accurate tools to identify individuals who could benefit from HIV prevention strategies, including PrEP.

Comparable to national-level estimates [24], we observed that 16% of individuals with a new HIV diagnosis used PrEP in the year preceding their diagnosis, of whom the majority still used PrEP in the previous 3 months. This observation suggests that certain individuals experience difficulty adhering to PrEP or continuing to use PrEP. Prior research has revealed several reasons for PrEP discontinuation, including lower perceived HIV risk, costs, competing life or health concerns, or scheduling challenges [25-28]. Interventions that could alleviate these barriers (such as improved guidance around stopping and restarting PrEP, online PrEP care, less frequent PrEP monitoring, and community-based care) should be offered to PrEP users who may benefit from it. The finding that the majority (60%) had used PrEP according to an event-driven regimen—while only 37% of PrEP users at the PHSA chose this regimen [29]—also implies a need to more thoroughly understand the real-world challenges and facilitators to using event-driven PrEP effectively. We observed that 20 individuals, most of whom were new to the PHSA, were diagnosed with HIV at a PrEP intake visit. This observation implies that facilitating timely PrEP uptake is crucial. This is corroborated by a previous qualitative study from the Netherlands in which delayed PrEP initiation (resulting from NPP waitlists, costs of PrEP care for the user, and limited PrEP-related knowledge among some general practitioners) was identified as a crucial missed opportunity for HIV prevention [30]. Furthermore, entry into PrEP care itself can be used as an important tool for testing, diagnosing and linkage to HIV care for those who are unaware of their status.

Our study described a large dataset of individuals diagnosed with HIV, including MSM and other key populations, before and during the expansion of PrEP access through the NPP. Nevertheless, there were some limitations. Firstly, we used only data from the PHSA; results might not be representative for other settings in the Netherlands [31]. Secondly, heterosexual cisgender men and cisgender women older than 25 years may have been underrepresented as they fall outside the PHSA’s demographic target population. Thirdly, there are services in Amsterdam dedicated to sexual healthcare for migrants and sex workers; individuals accessing these services might not be included in our analysis. Fourthly, detailed data on duration of PrEP use, current PrEP use and adherence were not collected; when analysing prior PrEP use, we were thus unable to distinguish between discontinued use and suboptimal adherence. Fifthly, prior PrEP use was only evaluated among individuals who had previously tested for HIV. As such, we may have underestimated the number of TGD persons, cisgender women and cisgender heterosexual men who used PrEP before their HIV diagnosis – although Dutch monitoring reports do indicate an extremely low PrEP uptake in these populations [7]. Sixthly, data were from a convenience sample, and some levels of determinants pertained to few numbers of individuals. Some results of the multivariable analysis could have consequently lacked statistical power. Finally, this study used cross-sectional data over time for a time-series analysis. Although this design allows for inference on the shift of sociodemographic and behavioural characteristics of those with HIV diagnoses, we cannot examine the sexual behaviours and PrEP use changes leading up to HIV diagnoses. These data would arguably be more informative for identifying the time point at which prevention efforts should be focused.

## Conclusion

As PrEP became implemented and integrated into sexual healthcare services through the NPP, a shift occurred in the population that remains at risk for HIV in Amsterdam. The increase in the number and proportion of HIV diagnoses from sex workers and TGD persons stresses the need for tailored and integrated services in the era of PrEP, while simultaneously highlighting successes in improved HIV testing in these groups. Most individuals received their HIV diagnosis at their first-ever visit to the PHSA, suggesting missed opportunities for earlier engagement. The reach and accessibility of HIV prevention, including PrEP services, should be expanded.

## Data Availability

Data are available from the corresponding author upon reasonable request.

## Supplementary Data

668000 Supplement
