# Supplementary material for: Characterising individuals with newly diagnosed HIV before versus during the era of PrEP: a descriptive time-series analysis of data from a sexual health centre in Amsterdam, the Netherlands, 2015 to 2024
Source: Euro Surveill. 2026 Jun 11;31(23):2500769. doi: 10.2807/1560-7917.ES.2026.31.23.2500769 (PMC13263649; doi:10.2807/1560-7917.ES.2026.31.23.2500769)
Supplement: Supplement [file 25-00769_WIJSTMA_Supplement.pdf]

This supplementary material is hosted by *Eurosurveillance* as supporting information alongside the article *"Characterizing individuals with newly diagnosed HIV during and before the era of PrEP: a descriptive time-series analysis of data from a sexual health center in Amsterdam, the Netherlands"*, on behalf of the authors, who remain responsible for the accuracy and appropriateness of the content. The same standards for ethics, copyright, attributions and permissions as for the article apply. Supplements are not edited by *Eurosurveillance* and the journal is not responsible for the maintenance of any links or email addresses provided therein.

Supplementary Figure S1.
Intersectionality of gender and sexuality group, country of birth, and engagement in sex work among individuals newly diagnosed with HIV at the Public Health Service of Amsterdam (1 January 2015-30 June 2024)

Supplementary Table S1.
Time since last negative HIV test* among individuals diagnosed with HIV at the Public Health Service of Amsterdam (2015-2024)

Supplementary Table S2.
Number of HIV tests and individuals tested for HIV per year, stratified by sociodemographic characteristics (Public Health Service of Amsterdam, 1 January 2015-30 June 2024)

Supplementary Table S3.
Number of HIV tests and individuals tested for HIV before and during the NPP, stratified by sociodemographic characteristics (Public Health Service of Amsterdam, 1 January 2015-30 June 2024)

Supplementary Figure S2.
Number of sex workers tested for HIV per year, stratified by ethnicity (Public Health Service of Amsterdam, 2015-2024)

Supplementary Table S4.
Sensitivity analysis on determinants of HIV diagnosis during versus before the national PrEP program, including condomless anal sex with a casual partner

Supplementary Figure S1. Intersectionality of gender and sexuality group, country of birth, and engagement in sex work among individuals newly diagnosed with HIV at the Public Health Service of Amsterdam (1 January 2015-30 June 2024)

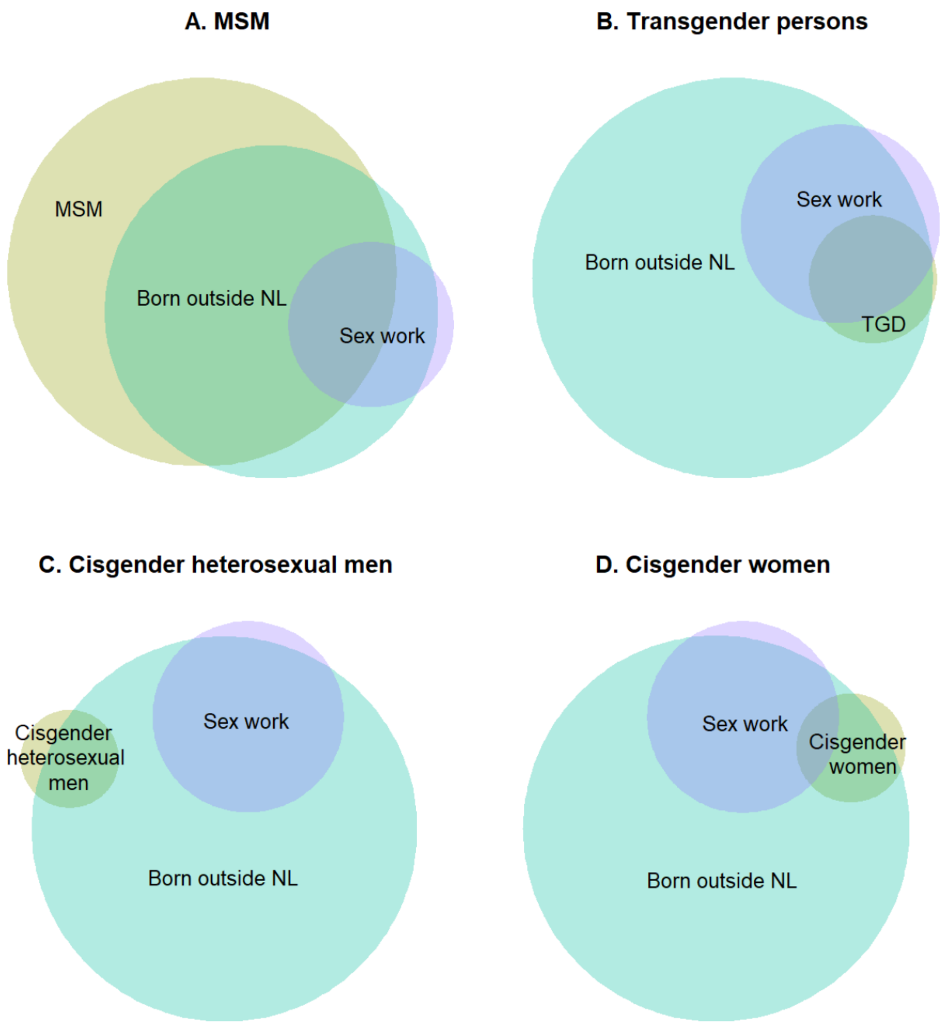

Abbreviations: MSM, men who have sex with men; NL, the Netherlands; TGD, transgender or gender-diverse; Panel A depicts intersectionality being MSM (n=536), doing sex work, and country of birth (CoB). Panel B depicts intersectionality of being TGD (n=42), sex work, and CoB. Panel C depicts intersectionality of being a cisgender heterosexual man (n=26), sex work, and CoB. Panel D depicts intersectionality of being a cisgender woman (n=33), sex work, and CoB.

Supplementary Table S1. Time since last negative HIV test* among individuals diagnosed with HIV at the Public Health Service of Amsterdam (2015-2024)

	Total (n=647)		Before NPP (n=425)		During NPP (n=222)		
	n	%	n	%	n	%	*p*-value†
Total							0.020
< 1 year	253	39.1%	184	43.3%	69	31.1%	
1-2 years	159	24.6%	97	22.8%	62	29.9%	
>2 years	143	22.1%	93	21.9%	50	22.5%	
No prior HIV test	74	11.4%	42	9.9%	32	14.4%	
Unknown	18	2.8%	9	2.1%	9	4.1%	
Sex workers							0.86
Total	99	100%	28	100%	71	100%	
< 1 year	31	31.3%	8	28.9%	23	32.4%	
1-2 years	30	30.3%	9	32.1%	21	29.6%	
>2 years	27	27.3%	7	25.0%	20	28.2%	
No prior HIV test	10	10.1%	4	14.3%	6	8.5%	
Unknown	1	1.0%	0	0.0%	1	1.4%	
TGD persons							0.035
Total	42	100%	5	100%	37	100%	
< 1 year	13	31.0%	1	20.0%	12	32.4%	
1-2 years	15	35.7%	3	60.0%	12	32.4%	
>2 years	11	26.2%	0	0.0%	11	29.7%	
No prior HIV test	2	4.8%	0	0.0%	2	5.4%	
Unknown	1	2.4%	1	20.0%	0	0.0%	
Born outside the Netherlands							0.62
Total	411	100%	248	100%	163	100%	
< 1 year	137	33.3%	90	36.3%	47	28.8%	
1-2 years	112	27.3%	63	25.4%	49	30.1%	
>2 years	93	22.6%	55	22.2%	38	23.3%	
No prior HIV test	55	13.4%	32	12.9%	23	14.1%	
Unknown	14	3.7%	8	3.2%	6	3.7%	

*Time since the last negative HIV test was based on visit history in case of a prior PHSA visit, supplemented by self-reported data.

†Overall p-value from Pearson's χ^2 test or Fisher's exact test.

Abbreviations: HIV, human immunodeficiency virus; NPP, national pre-exposure prophylaxis programme; TGD, transgender and gender diverse.

Supplementary Table S2. Number of HIV tests and individuals tested for HIV per year, stratified by sociodemographic characteristics (Public Health Service of Amsterdam, 1 January 2015-30 June 2024)

	**Total**		**MSM**		**TGD persons**		**Cisgender heterosexual men**		**Cisgender women**		**Non-Dutch ethnicity***		**Sex worker**		**Sex worker with Central/South American ethnicity***	
**year**	No. Tests	No. clients tested	No. Tests	No. clients tested	No. Tests	No. clients tested	No. Tests	No. clients tested	No. Tests	No. clients tested	No. Tests	No. clients tested	No. Tests	No. clients tested	No. Tests	No. clients tested
**2015**	29506	23118	9934	6677	1	1	7345	6445	11977	9949	14269	11094	2106	1227	307	192
**2016**	30844	22805	12630	7433	3	1	7173	6275	10853	9028	14549	10754	2115	1240	293	181
**2017**	32560	23363	14607	8246	190	115	7159	6239	10544	8743	15847	11449	2341	1341	370	225
**2018**	34746	23955	17129	9247	230	145	6735	5850	10600	8693	17587	12229	2449	1378	370	218
**2019**	32470	21297	18563	9647	318	189	5020	4418	8546	7034	16849	11339	2431	1411	436	277
**2020**	27916	17853	16926	8441	393	204	3634	3245	6955	5960	14597	9400	1929	1174	469	278
**2021**	35568	21295	21369	9506	670	334	4676	4083	8847	7369	18769	11087	2234	1245	621	341
**2022**	41515	24444	25296	11135	885	459	5477	4704	9850	8143	22682	13211	2334	1320	722	407
**2023**	40141	23765	25444	11602	1022	544	4931	4314	8744	7305	22558	13231	2441	1344	780	432
**2024****	17851	13242	12064	7973	639	463	1837	1730	3311	3076	10403	7663	1372	1017	433	334

Abbreviations: MSM. Men who have sex with men; TGD, transgender or gender-diverse; HIV, human immunodeficiency virus;
*Ethnicity was based on the country of birth of the client and their parent(s).
**Data up to 30 June 2024

Supplementary Table S3. Number of HIV tests and individuals tested for HIV before and during the NPP, stratified by sociodemographic characteristics (Public Health Service of Amsterdam, 1 January 2015-30 June 2024)

	Before NPP[§]		During NPP[§]	
	No. HIV tests	No. individuals tested	No. HIV tests	No. individuals tested
**Total**	147283	69051	175834	65363
**MSM**	64910	18692	109052	23465
**TGD persons**	592	170	3759	1109
**Cisgender heterosexual men**	31745	20693	22242	15234
**Cisgender women**	49476	29397	40751	25540
**Non-Dutch ethnicity**	72257	34019	95853	35585
**Sex worker**	10453	3145	11299	3489
**Sex worker with Central/South American ethnicity**	1575	489	3226	1073

Abbreviations: NPP, national PrEP pilot; MSM. Men who have sex with men; TGD, transgender or gender-diverse; HIV, human immunodeficiency virus;

[§]before NPP refers the period between 1 January 2015 and 31 July 2019, and during NPP refers to the period between 1 August 2019 and 30 June 2024.

*Ethnicity was based on the country of birth of the client and their parent(s).

Supplementary Figure S2. Number of sex workers tested for HIV per year, stratified by ethnicity (Public Health Service of Amsterdam, 2015-2024)

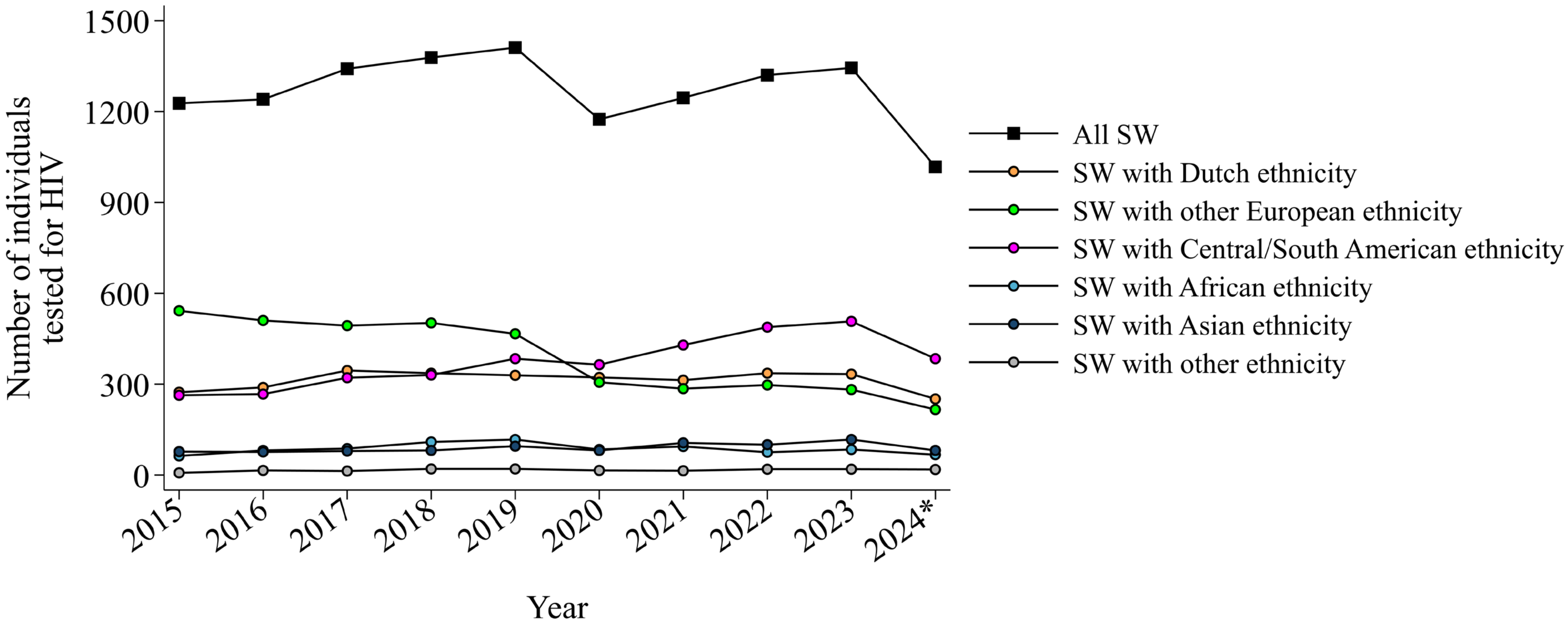

Abbreviations: SW, sex worker; HIV, human immunodeficiency virus;

Ethnicity was based on the country of birth of the client and their parent(s), and followed classification of Statistics Netherlands.

*In 2024, we included data up to 30 June 2024.

Supplementary Table S4. Sensitivity analysis on determinants of HIV diagnosis during versus before the national PrEP program, including condomless anal sex with a casual partner

	Univariable		Multivariable†	
	OR (95%CI)	p	aOR (95%CI)	p
Age (per year)	0.99 (0.97-1.00)	0.073	0.99 (0.97-1.01)	0.47
Sexual group¶		0.12‡		0.42‡
MSM	Ref.		Ref.	
TGD person	16.79 (6.49-43.49)		9.75 (3.09-30.79)	<0.001
Cisgender heterosexual man	1.42 (0.63-3.19)		3.14 (1.23-8.02)	0.016
Cisgender woman	0.73 (0.32-1.64)		1.02 (0.38-2.73)	0.97
Ethnicity°		0.21 ‡		0.45‡
European	Ref.		Ref.	
Central/South American	2.17 (1.50-3.15)	<0.001	1.28 (0.81-2.01)	0.29
African	0.82 (0.43-1.56)	0.54	0.68 (0.31-1.49)	0.34
Asian	1.47 (0.84-2.58)	0.18	1.34 (0.72-2.48)	0.36
Other/unknown	1.64 (0.52-5.17)	0.40	0.87 (0.23-3.28)	0.84
Tertiary education level	0.80 (0.57-1.12)	0.20	1.42 (0.94-2.16)	0.098
Any condomless anal sex with a casual partner* (yes vs. no)	2.26 (1.62-3.14)	<0.001	2.82 (1.91-4.18)	<0.001
Sex work* (yes vs. no)	6.58 (4.08-10.61)	<0.001	4.90 (2.68-8.96)	<0.001
Any STI diagnosed at HIV diagnosis visit (yes vs. no)	0.78 (0.56-1.09)	0.15	0.71 (0.48-1.04)	0.079

All parameter estimates were calculated using logistic regression with multiple imputation.

¶Ten cisgender men without available information on sexual preference were assumed to be MSM (4/10 were previously diagnosed with a rectal STI at the PHSA).

°Based on country of birth of the individual and their parents, following classification of Statistics Netherlands. The 'other' category includes people with North American and Oceanian ethnicity.

*Refers to six months prior to the visit.

†All odds ratios are adjusted by the variables presented in the table.

‡Overall *p*-value.

Abbreviations: aOR, adjusted odds ratio; CI, confidence interval; HIV, human immunodeficiency virus; OR, odds ratio; MSM, men who have sex with men; STI, sexually transmitted infection; TGD, transgender or gender-diverse.
